# Monoterpenoid Glycosides from the Leaves of *Ligustrum robustum* and Their Bioactivities (II)

**DOI:** 10.3390/molecules28217274

**Published:** 2023-10-26

**Authors:** Shi-Hui Lu, Xiu-Xia Li, Hao-Jiang Zuo, Wei-Neng Li, Jia-Ping Pan, Jing Huang

**Affiliations:** 1 College of Pharmacy, Youjiang Medical University for Nationalities, Baise 533000, China; laoli2299@163.com (W.-N.L.); 18778688727@163.com (J.-P.P.); 2 Guangxi Database Construction and Application Engineering Research Center for in Tracorporal Pharmacochemistry of TCM, Baise 533000, China; 3 Key Laboratory of Youjiang Basin Characteristic Ethnic Medicine Research in Guangxi, Baise 533000, China; 4 Nursing School, Youjiang Medical University for Nationalities, Baise 533000, China; axia-883333@163.com; 5 Department of Laboratory Science of Public Health, West China School of Public Health, Sichuan University, Chengdu 610041, China; zuohaojiang@scu.edu.cn; 6 Key Laboratory of Drug Targeting, Ministry of Education, West China School of Pharmacy, Sichuan University, Chengdu 610041, China

**Keywords:** *Ligustrum robustum*, monoterpenoid glycoside, FAS, α-glucosidase, antioxidant, anti-diabetes

## Abstract

*Ligustrum robustum* has been not only used as a heat-clearing and detoxicating functional tea (Ku-Ding-Cha) but also consumed as a hypotensive, anti-diabetic, and weight-reducing folk medicine. From the leaves of *L. robustum*, ten new monoterpenoid glycosides named ligurobustosides T_10_ (**1a**), T_11_ (**1b**), T_12_ (**2a**), T_13_ (**2b**), T_14_ (**3a**), T_15_ (**3b**), F_1_ (**4b**), T_16_ (**5a**), T_17_ (**5b**), and E_1_ (**6b**), together with five known ones (**4a**, **6a**, **7**, **8a**, **8b**), were separated and identified using the spectroscopic method and chemical method in this research. The results of biological tests exhibited that the fatty acid synthase (FAS) inhibitory action of compound **5** (IC_50_: 4.38 ± 0.11 μM) was as strong as orlistat (IC_50_: 4.46 ± 0.13 μM), a positive control; the α-glucosidase inhibitory actions of compounds **1**–**4** and **7**–**8**, and the α-amylase inhibitory actions of compounds **1**–**8** were medium; the ABTS radical scavenging capacities of compounds **1**–**3** and **5**–**8** (IC_50_: 6.27 ± 0.23 ~ 8.59 ± 0.09 μM) were stronger than l-(+)-ascorbic acid (IC_50_: 10.06 ± 0.19 μM) served as a positive control. This research offered a theoretical foundation for the leaves of *L. robustum* to prevent diabetes and its complications.

## 1. Introduction

Diabetes, which affects about 10.5% of the population in the world, is a chronic metabolic disease with the characteristic of hyperglycemia, and its complications, such as diabetic nephropathy, neuropathy, and cardiovascular diseases, result in high morbidity and mortality [1]. Present anti-diabetic agents, including insulin, metformin, α-glucosidase inhibitors acarbose and *N*-substituted iminosugar *C*-glycoside [2], can deal with hyperglycemia, whereas their function to prevent the complications of diabetes is not ideal. Recent research [3] indicated that antioxidant natural ingredients with inhibitory actions on fatty acid synthase (FAS), α-glucosidase, and α-amylase might be a novel resource for preventing diabetes and its complications.

**Figure 1:**
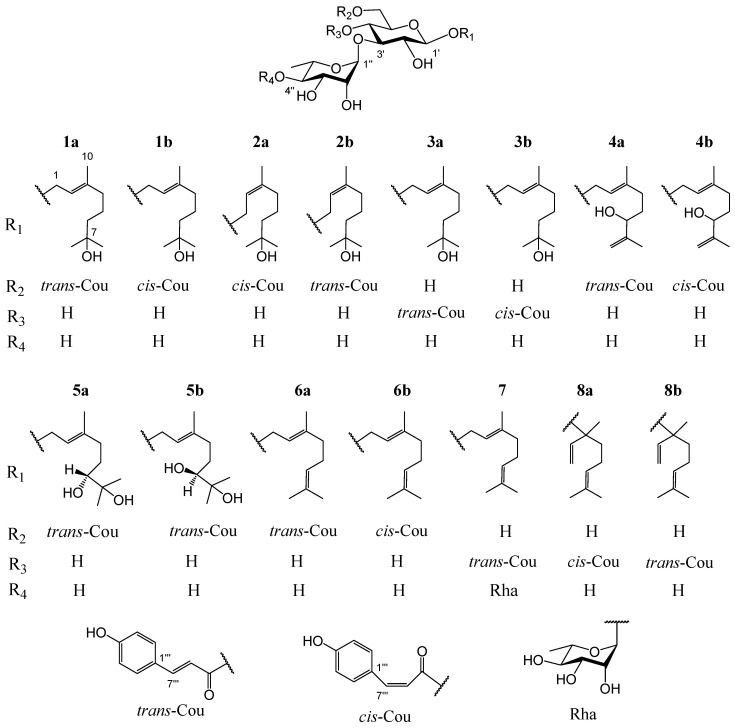
Structures of compounds **1**–**8** from the leaves of *L. robustum*.

*Ligustrum robustum* (Roxb.) Blume, a shrub of the Oleaceae family, is widespread in Southwest China, Vietnam, Burma, Cambodia, and India [3]. The leaves of *L. robustum*, containing 3.96–3.99 mg·g^−1^ *trans*-*p*-hydroxycinnamic acid and 202.6–210.6 mg·g^−1^ total *trans*-*p*-hydroxycinnamic acid esters [4], have been not only used as a heat-clearing and detoxicating functional tea (Ku-Ding-Cha) but also consumed as a hypotensive, anti-diabetic, and weight-reducing folk medicine [3]. In the preceding researches on *L. robustum* [3,4,5,6,7,8,9,10,11,12,13,14,15], about 90 chemical ingredients (of which 34 ingredients belonged to *trans*-*p*-hydroxycinnamic acid esters) involving phenylmethanoid glycosides, phenylethanoid glycosides, monoterpenoid glycosides, iridoid glycosides, butenol glycosides, hexenol glycosides, lignan glycosides, flavonoid glycosides, triterpenoids, sugar esters, and other compounds were separated and elucidated. At the same time, the antioxidant capacities and inhibitory actions of FAS, α-glucosidase, and α-amylase of many constituents were reported. In order to elucidate more *trans*-*p*-hydroxycinnamic acid esters and discover more functional ingredients for preventing diabetes and its complications, the study on the chemical compositions from the leaves of *L. robustum* and their bioactivities, which had been performed preliminary [3,15], was ulteriorly carried out. In this investigation, ten new monoterpenoid glycosides named ligurobustosides T_10_ (**1a**), T_11_ (**1b**), T_12_ (**2a**), T_13_ (**2b**), T_14_ (**3a**), T_15_ (**3b**), F_1_ (**4b**), T_16_ (**5a**), T_17_ (**5b**), and E_1_ (**6b**), along with five reported ones (**4a**, **6a**, **7**, **8a**, **8b**) (Figure 1), were separated and elucidated from the leaves of *L. robustum*. This article discusses the separation and elucidation of compounds **1**–**8** while reporting their inhibitory actions on FAS, α-glucosidase, α-amylase, and their antioxidant capacities.

## 2. Results and Discussion

### 2.1. Identification of Compounds **1**–**8**

**Figure 2:**
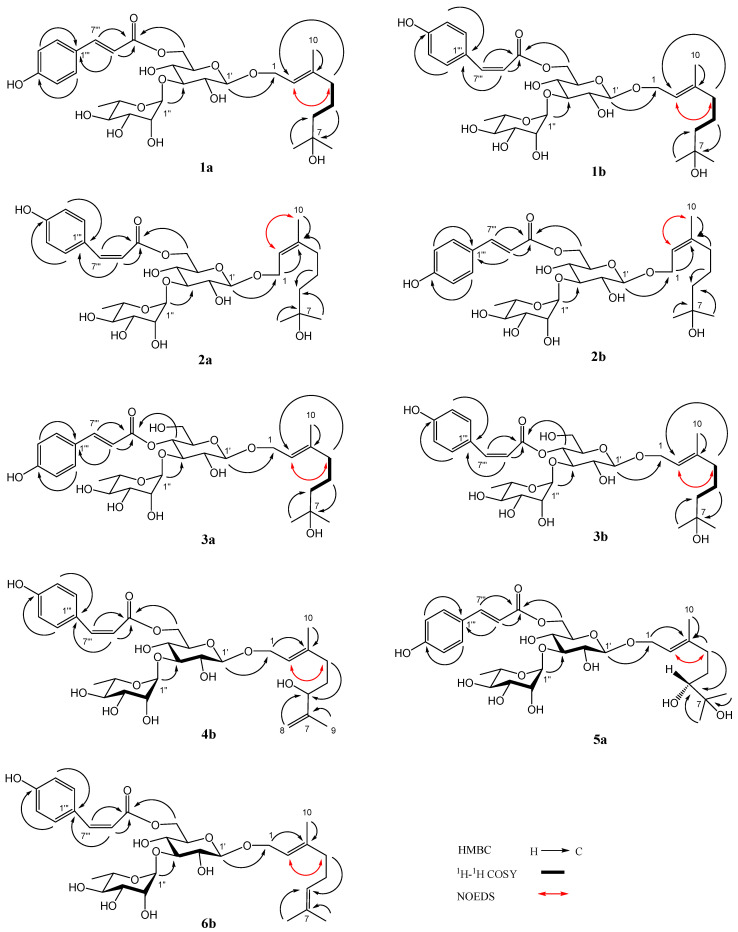
Key HMBC, ^1^H-^1^H COSY, and NOEDS correlations of compounds **1**–**6**.

Compound **1** was a yellowish amorphous powder. The HRESIMS of compound **1** showed a base peak at *m*/*z* 649.2836 [M + Na]^+^ (calculated for C_31_H_46_NaO_13_, 649.2836), confirming that its molecular formula was C_31_H_46_O_13_, which contains nine degrees of unsaturation. The IR spectrum of **1** suggested the presence of hydroxy (3366 cm^−1^), carbonyl (1693 cm^−1^), double bond (1632 cm^−1^), and phenyl (1604, 1515, 1447 cm^−1^). Two stereoisomers, **1a** and **1b** (3:1), were observed in the NMR spectra of compound **1**. The ^1^H NMR spectrum of **1a** (Table 1) indicated the presence of a *trans*-*p*-coumaroyl [*δ*_H_ 6.81, 7.45 (2 H each, d, *J* = 8.4 Hz), 6.35, 7.64 (1 H each, d, *J* = 16.2 Hz)], two monosaccharide residues [*δ*_H_ 4.32 (1 H, d, *J* = 7.8 Hz), 5.18 (1 H, d, *J* = 1.8 Hz), 1.25 (3 H, d, *J* = 6.6 Hz)], and a monoterpenoid aglycone [*δ*_H_ 5.37 (1 H, t, *J* = 7.2 Hz), 4.26, 4.27 (1 H each, d, *J* = 7.2 Hz), 2.02 (2 H, t, *J* = 7.2 Hz), 1.40, 1.48 (2 H each, m), 1.15, 1.15, 1.66 (3 H each, s)]. The ^13^C NMR spectrum of **1a** (Table 2) also confirmed the presence of a *trans*-*p*-coumaroyl (*δ*_C_ 114.9–169.0), two monosaccharide residues (*δ*_C_ 17.9–102.7), and a monoterpenoid aglycone (*δ*_C_ 16.4–142.4). The above ^1^H and ^13^C NMR features of **1a** were closely related to those of (2*E*,5*E*)-1-(1-hydroxy-7-methoxy-3,7-dimethyl-octa-2,5-dienyl)-(3-*O*-α-l-rhamnopyranosyl)-(6-*O*-*trans*-*p*-coumaroyl)-*O*-*β*-d-glucopyranoside (ligurobustoside T_8_) [15], except that the C-5 double bond and C-7 methoxy in ligurobustoside T_8_ were replaced by a single bond and a hydroxy in **1a**. The ^1^H-^1^H COSY of **1a** (Figure 2) showed the correlations between H-4, 6 of aglycone (*δ*_H_ 2.02, 1.40), and H-5 of aglycone (*δ*_H_ 1.48), suggesting that there was a single bond at C-5 of **1a**. The NOEDS of **1a** (Figure 2) exhibited a correlation between H-2 of aglycone (*δ*_H_ 5.37) and H-4 of aglycone (*δ*_H_ 2.02), confirming that the C-2 double bond was *E*-configuration. Combined with the HMBC spectrum of **1a** (Figure 2), the aglycone of **1a** was determined to be (*E*)-3,7-dimethyl-2-octene-1,7-diol. In addition, d-glucose and l-rhamnose, which were identified by TLC, were obtained from the acid hydrolysis experiment of compound **1**. The long-distance correlations between H-1′ of Glc (*δ*_H_ 4.32) and C-1 of aglycone (*δ*_C_ 66.2), between H-1″ of Rha (*δ*_H_ 5.18) and C-3′ of Glc (*δ*_C_ 84.0), and between H-6′ of Glc (*δ*_H_ 4.36, 4.51) and carbonyl of Cou (*δ*_C_ 169.0) were displayed in the HMBC spectrum of **1a** (Figure 2). ^1^H-^1^H COSY, HSQC, HMBC, and NOEDS (Figure S1) were applied to the assignment of the ^1^H and ^13^C NMR signals of compound **1**. Based on the above evidences, **1a** was identified as (*E*)-7-hydroxy-3,7-dimethyl-2-octen-1-yl 3-*O*-(α-l-rhamnopyranosyl)-6-*O*-(*trans*-*p*-coumaroyl)-*O*-*β*-d-glucopyranoside. It is a novel monoterpenoid glycoside named ligurobustoside T_10_.

The NMR characteristics of **1b** (Table 1 and Table 2) were similar to those of **1a**, except that the *cis*-*p*-coumaroyl [*δ*_H_ 6.74, 7.66 (2 H each, d, *J* = 8.4 Hz), 5.76, 6.87 (1 H each, d, *J* = 12.6 Hz)] in **1b** took the place of the *trans*-*p*-coumaroyl in **1a**. The long-distance correlations between H-1′ of Glc (*δ*_H_ 4.26) and C-1 of aglycone (*δ*_C_ 66.2), between H-1″ of Rha (*δ*_H_ 5.15) and C-3′ of Glc (*δ*_C_ 84.5), and between H-6′ of Glc (*δ*_H_ 4.29, 4.48) and carbonyl of Cou (*δ*_C_ 168.2) were shown in the HMBC spectrum of **1b** (Figure 2). Thus, compound **1b** was identified as (*E*)-7-hydroxy-3,7-dimethyl-2-octen-1-yl 3-*O*-(α-l-rhamnopyranosyl)-6-*O*-(*cis*-*p*-coumaroyl)-*O*-*β*-d-glucopyranoside. It is a new monoterpenoid glycoside named ligurobustoside T_11_. In conclusion, compound **1** is made up of ligurobustosides T_10_ and T_11_.

Compound **2** was obtained as a white amorphous powder. The molecular formula C_31_H_46_O_13_ containing nine degrees of unsaturation was determined by HRESIMS at *m*/*z* 649.2836 [M + Na]^+^ (calculated for C_31_H_46_NaO_13_, 649.2836). The IR spectrum of **2** indicated the existence of hydroxy (3366 cm^−1^), carbonyl (1693 cm^−1^), double bond (1632 cm^−1^), and phenyl (1604, 1515, 1447 cm^−1^). Two stereoisomers, **2a** and **2b** (3:1), were displayed in the NMR spectra of compound **2**. The ^1^H and ^13^C NMR characteristics of **2a** (Table 1 and Table 2) were very similar to those of **1b**, except for the configuration of the C-2 double bond of aglycone. The correlation between H-2 of aglycone (*δ*_H_ 5.35) and H-10 of aglycone (*δ*_H_ 1.64) was revealed in the NOEDS of **2a** (Figure 2), suggesting that the C-2 double bond was *Z*-configuration. d-glucose and l-rhamnose identified by TLC were obtained from the acid hydrolysis experiment of compound **2**. Additionally, the long-distance correlations between H-1′ of Glc (*δ*_H_ 4.28) and C-1 of aglycone (*δ*_C_ 66.2), between H-1″ of Rha (*δ*_H_ 5.15) and C-3′ of Glc (*δ*_C_ 84.1), and between H-6′ of Glc (*δ*_H_ 4.29, 4.49) and carbonyl of Cou (*δ*_C_ 168.1) were displayed in the HMBC spectrum of **2a** (Figure 2). HMBC and NOEDS (Figure S2) were used to assign the ^1^H and ^13^C NMR signals of compound **2**. Therefore, **2a** was identified as (*Z*)-7-hydroxy-3,7-dimethyl-2-octen-1-yl 3-*O*-(α-l-rhamnopyranosyl)-6-*O*-(*cis*-*p*-coumaroyl)-*O*-*β*-d-glucopyranoside. It is a new monoterpenoid glycoside named ligurobustoside T_12_.

The NMR features of **2b** (Table 1 and Table 2) were closely related to those of **2a**, except that the *trans*-*p*-coumaroyl [*δ*_H_ 6.81, 7.46 (2 H each, d, *J* = 8.8 Hz), 6.31, 7.64 (1 H each, d, *J* = 16.0 Hz)] in **2b** took the place of *cis*-*p*-coumaroyl [*δ*_H_ 6.76, 7.65 (2 H each, d, *J* = 8.8 Hz), 5.80, 6.88 (1 H each, d, *J* = 12.8 Hz)] in **2a**. From the HMBC spectrum of **2b** (Figure 2), the long-distance correlations between H-1′ of Glc (*δ*_H_ 4.32) and C-1 of aglycone (*δ*_C_ 66.2), between H-1″ of Rha (*δ*_H_ 5.17) and C-3′ of Glc (*δ*_C_ 84.0), and between H-6′ of Glc (*δ*_H_ 4.35, 4.50) and carbonyl of Cou (*δ*_C_ 169.3) were observed. Thus, compound **2b** was confirmed as (*Z*)-7-hydroxy-3,7-dimethyl-2-octen-1-yl 3-*O*-(α-l-rhamnopyranosyl)-6-*O*-(*trans*-*p*-coumaroyl)-*O*-*β*-d-glucopyranoside, which is a novel monoterpenoid glycoside named ligurobustoside T_13_. To sum up, compound **2** is composed of ligurobustosides T_12_ and T_13_.

Compound **3**, a white amorphous powder, was determined to be C_31_H_46_O_13_ by HRESIMS (*m*/*z* 649.2834 [M + Na]^+^, calculated 649.2836 for C_31_H_46_NaO_13_), which contains nine degrees of unsaturation. The hydroxy (3391 cm^−1^), carbonyl (1698 cm^−1^), double bond (1632 cm^−1^), and phenyl (1605, 1515 cm^−1^) were observed in the IR spectrum of **3**. Two stereoisomers, **3a** and **3b** (3:1), were exhibited in the NMR spectra of compound **3**. The ^1^H and ^13^C NMR characteristics of **3a** (Table 1 and Table 2) were closely related to those of **1a**, except for a *trans*-*p*-coumaroyl linking with different positions. The correlation between H-2 of aglycone (*δ*_H_ 5.40) and H-4 of aglycone (*δ*_H_ 2.06) was shown in the NOEDS of **3a** (Figure 2), affirming further that the C-2 double bond was *E*-configuration. d-glucose and l-rhamnose, confirmed by TLC, were offered from the acid hydrolysis experiment of compound **3**. Furthermore, the long-distance correlations between H-1′ of Glc (*δ*_H_ 4.38) and C-1 of aglycone (*δ*_C_ 66.5), between H-1″ of Rha (*δ*_H_ 5.19) and C-3′ of Glc (*δ*_C_ 81.6), and between H-4′ of Glc (*δ*_H_ 4.92) and carbonyl of Cou (*δ*_C_ 168.3) were revealed in the HMBC spectrum of **3a** (Figure 2). ^1^H-^1^H COSY, HSQC, HMBC, and NOEDS (Figure S3) were applied to the assignment of the ^1^H and ^13^C NMR signals of compound **3**. Consequently, compound **3a** was confirmed to be (*E*)-7-hydroxy-3,7-dimethyl-2-octen-1-yl 3-*O*-(α-l-rhamnopyranosyl)-4-*O*-(*trans*-*p*-coumaroyl)-*O*-*β*-d-glucopyranoside, which is a new monoterpenoid glycoside named ligurobustoside T_14_.

The NMR features of **3b** (Table 1 and Table 2) were very close to those of **3a**, the main difference was that the *cis*-*p*-coumaroyl [*δ*_H_ 6.76, 7.62 (2 H each, d, *J* = 8.4 Hz), 5.79, 6.95 (1 H each, d, *J* = 12.8 Hz)] in **3b** took the place of the *trans*-*p*-coumaroyl [*δ*_H_ 6.81, 7.47 (2 H each, d, *J* = 8.4 Hz), 6.34, 7.66 (1 H each, d, *J* = 16.0 Hz)] in **3a**. The long-distance correlations between H-1′ of Glc (*δ*_H_ 4.33) and C-1 of aglycone (*δ*_C_ 66.5), between H-1″ of Rha (*δ*_H_ 5.16) and C-3′ of Glc (*δ*_C_ 81.9), and between H-4′ of Glc (*δ*_H_ 4.86) and carbonyl of Cou (*δ*_C_ 166.9) were exhibited in the HMBC spectrum of **3b** (Figure 2). Therefore, the structure of compound **3b** was identified as (*E*)-7-hydroxy-3,7-dimethyl-2-octen-1-yl 3-*O*-(α-l-rhamnopyranosyl)-4-*O*-(*cis*-*p*-coumaroyl)-*O*-*β*-d-glucopyranoside. It is a new monoterpenoid glycoside named ligurobustoside T_15_. In sum, compound **3** is made up of ligurobustosides T_14_ and T_15_.

Compound **4**, a white amorphous powder, was analyzed to be C_31_H_44_O_13_ by HRESIMS (*m*/*z* 647.2684 [M + Na]^+^, calculated 647.2680 for C_31_H_44_NaO_13_), which contains ten degrees of unsaturation. The hydroxy (3392 cm^−1^), carbonyl (1696 cm^−1^), double bond (1633 cm^−1^), and phenyl (1605, 1515 cm^−1^) were observed in the IR spectrum of **4**. Two stereoisomers, **4a** and **4b** (5:3), were shown in the NMR spectra of compound **4**. The ^1^H and ^13^C NMR data of **4a** (Supplementary Material S1) were consistent with those of (2*E*)-6-hydroxy-3,7-dimethyl-2,7-octadien-1-yl 3-*O*-(α-l-rhamnopyranosyl)-6-*O*-(*trans*-*p*-coumaroyl)-*O*-*β*-d-glucopyranoside (ligurobustoside F) [8]. The ^1^H and ^13^C NMR characteristics of **4b** (Table 1 and Table 2) were close to those of **4a**, the main difference was that the *cis*-*p*-coumaroyl [*δ*_H_ 6.74, 7.66 (2 H each, d, *J* = 8.4 Hz), 5.77, 6.87 (1 H each, d, *J* = 12.8 Hz)] in **4b** took the place of the *trans*-*p*-coumaroyl [*δ*_H_ 6.77, 7.44 (2 H each, d, *J* = 8.8 Hz), 6.32, 7.64 (1 H each, d, *J* = 16.0 Hz)] in 4**a**. The correlation between H-2 of aglycone (*δ*_H_ 5.38) and H-4 of aglycone (*δ*_H_ 2.07) was displayed in the NOEDS of **4b** (Figure 2), confirming further that the C-2 double bond was *E*-configuration. d-glucose and l-rhamnose identified by TLC were obtained from the acid hydrolysis experiment of compound **4**. Moreover, the long-distance correlations between H-1′ of Glc (*δ*_H_ 4.30) and C-1 of aglycone (*δ*_C_ 66.4), between H-1″ of Rha (*δ*_H_ 5.15) and C-3′ of Glc (*δ*_C_ 84.0), and between H-6′ of Glc (*δ*_H_ 4.30, 4.49) and carbonyl of Cou (*δ*_C_ 168.2) were exhibited in the HMBC spectrum of **4b** (Figure 2). ^1^H-^1^H COSY, HSQC, HMBC, and NOEDS (Figure S4) were used to assign the ^1^H and ^13^C NMR signals of compound **4**. So, **4b** was elucidated to be (2*E*)-6-hydroxy-3,7-dimethyl-2,7-octadien-1-yl 3-*O*-(α-l-rhamnopyranosyl)-6-*O*-(*cis*-*p*-coumaroyl)-*O*-*β*-d-glucopyranoside. It is a novel monoterpenoid glycoside named ligurobustoside F_1_. To sum up, compound **4** is made up of ligurobustosides F and F_1_.

Compound **5** was acquired as a white amorphous powder. Its molecular formula C_31_H_46_O_14_, which contains nine degrees of unsaturation, was affirmed by HRESIMS at *m*/*z* 665.2790 [M + Na]^+^ (calculated for C_31_H_46_NaO_14_, 665.2785). The presence of hydroxy (3375 cm^−1^), carbonyl (1693 cm^−1^), double bond (1632 cm^−1^), and phenyl (1604, 1515, 1448 cm^−1^) was confirmed with the IR spectrum of **5**. Two stereoisomers, **5a** and **5b** (6:4), were revealed in the NMR spectra of compound **5**. The ^1^H NMR spectrum of **5a** (Table 1) affirmed the existence of a *trans*-*p*-coumaroyl [*δ*_H_ 6.81, 7.46 (2 H each, d, *J* = 8.4 Hz), 6.35, 7.64 (1 H each, d, *J* = 16.0 Hz)], two monosaccharide residues [*δ*_H_ 4.32 (1 H, d, *J* = 7.6 Hz), 5.17 (1 H, d, *J* = 2.0 Hz), 1.24 (3 H, d, *J* = 6.4 Hz)], and a monoterpenoid aglycone [*δ*_H_ 5.41 (1 H, t, *J* = 7.2 Hz), 4.26, 4.27 (1 H each, d, *J* = 7.2 Hz), 3.23 (1 H, dd, *J* = 10.4, 2.0 Hz), 1.33, 1.76, 2.08, 2.29 (1 H each, m), 1.12, 1.16, 1.68 (3 H each, s)]. The ^13^C NMR spectrum of **5a** (Table 2) also indicated the presence of a *trans*-*p*-coumaroyl (*δ*_C_ 114.9–169.1), two monosaccharide residues (*δ*_C_ 17.9–102.7), and a monoterpenoid aglycone (*δ*_C_ 16.5–142.5). The above ^1^H and ^13^C NMR characteristics of **5a** were close to those of (*E*)-6,7-dihydroxy-3,7-dimethyl-2-octen-1-yl 3-*O*-(α-l-rhamnopyranosyl)-4-*O*-(*cis*-*p*-coumaroyl)-*O*-*β*-d-glucopyranoside (ligurobustoside L) [9], except that the *cis*-*p*-coumaroyl in ligurobustoside L was taken in place of a *trans*-*p*-coumaroyl at a different position in **5a**. The correlation between H-2 of aglycone (*δ*_H_ 5.41) and H-4 of aglycone (*δ*_H_ 2.08, 2.29) was shown in the NOEDS of **5a** (Figure 2), suggesting that the C-2 double bond was *E*-configuration. d-glucose and l-rhamnose, which were confirmed by TLC, were obtained from the acid hydrolysis experiment of compound **5**. Moreover, the long-distance correlations between H-1′ of Glc (*δ*_H_ 4.32) and C-1 of aglycone (*δ*_C_ 66.2), between H-1″ of Rha (*δ*_H_ 5.17) and C-3′ of Glc (*δ*_C_ 84.0), and between H-6′ of Glc (*δ*_H_ 4.36, 4.51) and carbonyl of Cou (*δ*_C_ 169.1) were displayed in the HMBC spectrum of **5a** (Figure 2). ^1^H-^1^H COSY, HSQC, HMBC, and NOEDS (Figure S5) were applied to the assignment of the ^1^H and ^13^C NMR signals of **5**. Thus, the plane structure of **5a** was identified as (*E*)-6,7-dihydroxy-3,7-dimethyl-2-octen-1-yl 3-*O*-(α-l-rhamnopyranosyl)-6-*O*-(*trans*-*p*-coumaroyl)-*O*-*β*-d-glucopyranoside.

The NMR features of **5b** (Table 1 and Table 2) were very similar to those of **5a**, except for the C-6 configuration of aglycone. The enzymatic hydrolysis experiment of **5** gave (*E*,*R*)-3,7-dimethyl-oct-2-ene-1,6,7-triol and (*E*,*S*)-3,7-dimethyl-oct-2-ene-1,6,7-triol (6:4). Together with **5a**:**5b** = 6:4 observed in the ^1^H NMR spectrum of compound **5**, the C-6 configurations of the aglycones in **5a** and **5b** were affirmed to be 6*R* and 6*S*, respectively. Based on the above evidences, **5a** was identified as (2*E*,*6R*)-6,7-dihydroxy-3,7-dimethyl-2-octen-1-yl 3-*O*-(α-l-rhamnopyranosyl)-6-*O*-(*trans*-*p*-coumaroyl)-*O*-*β*-d-glucopyranoside, which is a new monoterpenoid glycoside named ligurobustoside T_16_; **5b** was identified as (2*E*,*6S*)-6,7-dihydroxy-3,7-dimethyl-2-octen-1-yl 3-*O*-(α-l-rhamnopyranosyl)-6-*O*-(*trans*-*p*-coumaroyl)-*O*-β-d-glucopyranoside, which is a new monoterpenoid glycoside named ligurobustoside T_17_. In conclusion, compound **5** is composed of ligurobustosides T_16_ and T_17_.

Compound **6**, a yellowish oil, was analyzed as C_31_H_44_O_12_ by HRESIMS (*m*/*z* 631.2728 [M + Na]^+^, calculated 631.2730 for C_31_H_44_NaO_12_), which contains ten degrees of unsaturation. The hydroxy (3391 cm^−1^), carbonyl (1694 cm^−1^), double bond (1633 cm^−1^), and phenyl (1605, 1515, 1444 cm^−1^) were observed in the IR spectrum of **6**. Two stereoisomers, **6a** and **6b** (5:1), were displayed in the NMR spectra of compound **6**. The ^1^H and ^13^C NMR data of **6a** (Supplementary Material S1) were consistent with those of geraniol 3-*O*-(α-l-rhamnopyranosyl)-6-*O*-(*trans*-*p*-coumaroyl)-*O*-*β*-d-glucopyranoside (ligurobustoside E) [8]. The ^1^H and ^13^C NMR features of **6b** (Table 1 and Table 2) were close to those of **6a**, except that the *cis*-*p*-coumaroyl [*δ*_H_ 6.76, 7.64 (2 H each, d, *J* = 8.4 Hz), 5.79, 6.87 (1 H each, d, *J* = 13.2 Hz)] in **6b** took the place of the *trans*-*p*-coumaroyl [*δ*_H_ 6.81, 7.46 (2 H each, d, *J* = 8.4 Hz), 6.35, 7.64 (1 H each, d, *J* = 16.2 Hz)] in **6a**. The correlation between H-2 of aglycone (*δ*_H_ 5.35) and H-4 of aglycone (*δ*_H_ 2.03) was shown in the NOEDS of **6b** (Figure 2), affirming further that the C-2 double bond was *E*-configuration. d-glucose and l-rhamnose identified by TLC were obtained from the acid hydrolysis experiment of compound **6**. In addition, the long-distance correlations between H-1′ of Glc (*δ*_H_ 4.28) and C-1 of aglycone (*δ*_C_ 66.2), between H-1″ of Rha (*δ*_H_ 5.15) and C-3′ of Glc (*δ*_C_ 84.0), and between H-6′ of Glc (*δ*_H_ 4.30, 4.45) and carbonyl of Cou (*δ*_C_ 168.1) were revealed in the HMBC spectrum of **6b** (Figure 2). ^1^H-^1^H COSY, HSQC, HMBC, and NOEDS (Figure S6) were used to assign the ^1^H and ^13^C NMR signals of compound **6**. Hence, **6b** was elucidated as (2*E*)-3,7-dimethyl-2,6-octadien-1-yl 3-*O*-(α-l-rhamnopyranosyl)-6-*O*-(*cis*-*p*-coumaroyl)-*O*-*β*-d-glucopyranoside. It is a novel monoterpenoid glycoside named ligurobustoside E_1_. To sum up, compound **6** is made up of ligurobustosides E and E_1_.

Compounds **7**–**8** (^1^H NMR, ^13^C NMR data see Supplementary Material S1) were confirmed to be known monoterpenoid glycosides ligurobustoside J (**7**) [8], lipedoside B-II (**8a**) [16], and lipedoside B-III (**8b**) [16], by comparison with the reported NMR information and 2D-NMR (^1^H-^1^H COSY, HSQC, and HMBC) experiments. Compound **8a** was separated from *L. robustum* for the first time.

### 2.2. The Bioactivities of Compounds **1**–**8**

The bioactivities of compounds **1**–**8** isolated from the leaves of *L. robustum*, including the inhibitory actions on FAS, α-glucosidase and α-amylase, and the antioxidant capacities, were measured. The results of the biological tests are listed in Table 3. As exhibited in Table 3, the FAS inhibitory action of compound **5** (IC_50_: 4.38 ± 0.11 μM) was as strong as orlistat (IC_50_: 4.46 ± 0.13 μM) applied as a positive control, whereas the FAS inhibitory actions of compounds **4**, **6**, and **8** (IC_50_: 6.78 ± 0.18~24.68 ± 0.27 μM) were weaker than orlistat; the α-glucosidase inhibitory actions of compounds **1**–**4** and **7**–**8** were medium and weaker than acarbose, a positive control; the α-amylase inhibitory actions of compounds **1**–**8** were medium and weaker than the positive control acarbose. Although the DPPH radical scavenging capacities of compounds **1**–**8** were not observed, the ABTS radical scavenging capacities of compounds **1**–**3** and **5**–**8** (IC_50_: 6.27 ± 0.23~8.59 ± 0.09 μM) were stronger than l-(+)-ascorbic acid (IC_50_: 10.06 ± 0.19 μM), which was used as a positive control.

Because antioxidant compositions with inhibitory actions on FAS, α-glucosidase, and α-amylase might be a novel resource for preventing diabetes and its complications [3], strong antioxidant compounds **1**–**8**, with strong or medium FAS, α-glucosidase, and α-amylase inhibitory actions, might be partial functional ingredients of *L. robustum* for preventing diabetes and its complications.

## 3. Materials and Methods

### 3.1. General Experimental Procedure

HRESIMS was measured using a Waters Q-TOF Premier mass spectrometer (Waters, Milford, MA, USA). The IR spectrum was recorded on a PerkinElmer Spectrum Two FT-IR spectrometer (PerkinElmer, Waltham, MA, USA). ^1^H NMR, ^13^C NMR, ^1^H-^1^H COSY, HSQC, HMBC, and NOEDS experiments were carried out on an Agilent 600/54 Premium Compact NMR spectrometer (Agilent, Santa Clara, CA, USA) or a Bruker Ascend^TM^ 400 NMR spectrometer (Bruker, Mannheim, Germany) with CD_3_OD as solvent at 25 °C, while tetramethylsilane (TMS) was used as an internal standard. Chemical shifts are expressed in *δ* (ppm), and coupling constants (*J*) are reported in Hz. The UV spectrum was performed with a UV2700 spectrophotometer (Shimadzu, Kyoto, Japan). The optical rotation value was examined on an AUTOPOL VI automatic polarimeter (Rudolph, Hackettstown, NJ, USA).

Column chromatography (CC) was carried out on polyamide (60–90 mesh, Jiangsu Changfeng Chemical Industry Co., Yangzhou, China), silica gel (SiO_2_: 200–300 mesh, Qingdao Ocean Chemical Industry Co., Qingdao, China), and MCI-gel CHP-20P (75–150 μm, Mitsubishi Chemical Co., Tokyo, Japan). Preparative HPLC was performed with a GL3000–300 mL system instrument (Chengdu Gelai Precision Instruments Co., Ltd., Chengdu, China) matching a C-18 column (5 μm, 50 × 450 mm), which was eluted with MeOH-H_2_O at 30 mL/min and monitored with a UV-3292 detector at 215 nm. TLC was executed on a precoated HPTLC Fertigplatten Kieselgel 60 F_254_ plate (Merck, Rahway, NJ, USA), which was sprayed with an α-naphthol-sulfuric acid solution or 10% sulfuric acid ethanolic solution and heated at 100–105 °C for 2–3 min. UV–vis absorbance was examined using a UV2700 spectrophotometer (Shimadzu, Kyoto, Japan) or a Spark 10M microplate reader (Tecan Trading Co. Ltd., Shanghai, China). NADPH and Ac-CoA were offered by Zeye Biochemical Co., Ltd. (Shanghai, China). Mal-CoA was obtained by Sigma-Aldrich (St. Louis, MO, USA). ABTS was supplied by Aladdin Industrial Co., Ltd. (Shanghai, China). DPPH was acquired from Macklin Biochemical Co., Ltd. (Shanghai, China).

### 3.2. Plant Material

The fresh leaves of *L. robustum* collected from Yibin City, China, were affirmed by Doctor Guo-Min Liu, Hainan University. A voucher sample (No. 201704lsh) of the leaves of *L. robustum* was preserved at the West China School of Pharmacy, Sichuan University.

### 3.3. Extraction and Separation

The fresh leaves of *L. robustum* were turned and heated at 115–120 °C for about 1 h, then powdered. The powdered leaves (7.0 kg) were extracted under reflux in a multi-function extractor with 70% ethanol (28 L × 1) for 2 h [15]. After filtration, the ethanol extract was condensed in vacuo to give a brownish-black paste (2.2 kg). The paste was dissolved with 95% ethanol (3 L), and then distilled water (3 L) was added to deposit the chlorophyll. After percolation, the filtrate was condensed in vacuo to acquire a brown residue (1.0 kg). The residue was chromatographed using a silica gel column, which was eluted with CH_2_Cl_2_-MeOH (10:0–0:10) to afford Fr. II (145 g) and three other fractions. Fr. II was chromatographed repeatedly on silica gel column (CH_2_Cl_2_-MeOH-H_2_O, 200:10:1–80:20:2; or EtOAc-MeOH-H_2_O, 100:4:2–100:6:2), and then separated by CC with polyamide (EtOH-H_2_O, 0:10–7:3) and MCI (MeOH-H_2_O, 1:9–7:3), and isolated finally by preparative HPLC (MeOH-H_2_O, 55:45–64:36) and silica gel column (EtOAc-MeOH-H_2_O, 100:2:1–100:6:2), to give compounds **1** (187.4 mg), **2** (17.6 mg), **3** (90.5 mg), **4** (4.2 mg), **5** (27.4 mg), **6** (169.1 mg), **7** (16.8 mg) and **8** (80.5 mg).

Compound **1**: yellowish amorphous powder. UV (MeOH) λ_max_: (log ε) 213 (4.1), 227 (4.2), 316 (4.4) nm; IR (film) ν_max_: 3366, 2926, 1693, 1632, 1604, 1515, 1447, 1268, 1166, 1038, 912, 834 cm^–1^; ^1^H NMR (CD_3_OD, 600 MHz) data, see Table 1; ^13^C NMR (CD_3_OD, 150 MHz) data, see Table 2; HRESIMS *m*/*z* 649.2836 [M + Na]^+^ (calculated for C_31_H_46_NaO_13_, 649.2836).

Compound **2**: white amorphous powder. UV (MeOH) λ_max_ (log ε): 213 (4.1), 227 (4.2), 316 (4.4) nm; IR (film) ν_max_: 3366, 2926, 1693, 1632, 1604, 1515, 1447, 1268, 1166, 1038, 912, 834 cm^–1^; ^1^H NMR (CD_3_OD, 400 MHz) data, see Table 1; ^13^C NMR (CD_3_OD, 150 MHz) data, see Table 2; HRESIMS *m*/*z* 649.2836 [M + Na]^+^ (calculated for C_31_H_46_NaO_13_, 649.2836).

Compound **3**: white amorphous powder. UV (MeOH) λ_max_ (log ε): 214 (4.1), 228 (4.2), 316 (4.4) nm; IR (film) ν_max_: 3391, 2935, 1698, 1632, 1605, 1515, 1263, 1169, 1041, 835 cm^–1^; ^1^H NMR (CD_3_OD, 400 MHz) data, see Table 1; ^13^C NMR (CD_3_OD, 100 MHz) data, see Table 2; HRESIMS *m*/*z* 649.2834 [M + Na]^+^ (calculated for C_31_H_46_NaO_13_, 649.2836).

Compound **4**: white amorphous powder. UV (MeOH) λ_max_ (log ε): 213 (4.1), 228 (4.2), 317 (4.4) nm; IR (film) ν_max_: 3392, 2926, 1696, 1633, 1605, 1515, 1263, 1169, 1040, 835 cm^–1^; ^1^H NMR (CD_3_OD, 400 MHz) data, see Table 1; ^13^C NMR (CD_3_OD, 150 MHz) data, see Table 2; HRESIMS *m*/*z* 647.2684 [M + Na]^+^ (calculated for C_31_H_44_NaO_13_, 647.2680).

Compound **5**: white amorphous powder. [α${]}_{D}^{23}$ −38.7 (*c* 0.55, MeOH); UV (MeOH) λ_max_ (log ε): 214 (4.1), 227 (4.2), 316 (4.4) nm; IR (film) ν_max_: 3375, 2925, 1693, 1632, 1604, 1515, 1448, 1262, 1170, 1070, 833 cm^–1^; ^1^H NMR (CD_3_OD, 400 MHz) data, see Table 1; ^13^C NMR (CD_3_OD, 150 MHz) data, see Table 2; HRESIMS *m*/*z* 665.2790 [M + Na]^+^ (calculated for C_31_H_46_NaO_14_, 665.2785).

Compound **6**: yellowish oil. [α${]}_{D}^{23}$ −66.2 (*c* 0.24, MeOH); UV (MeOH) λ_max_ (log ε): 214 (4.1), 227 (4.2), 316 (4.4) nm; IR (film) ν_max_: 3391, 2925, 1694, 1633, 1605, 1515, 1444, 1264, 1170, 1037, 832 cm^–1^; ^1^H NMR (CD_3_OD, 600 MHz) data, see Table 1; ^13^C NMR (CD_3_OD, 100 MHz) data, see Table 2; HRESIMS *m*/*z* 631.2728 [M + Na]^+^ (calculated for C_31_H_44_NaO_12_, 631.2730).

### 3.4. Acid Hydrolysis of Compounds **1**–**6**

Compounds **1**–**6** (2 mg, each) were hydrolyzed with 1 M H_2_SO_4_ aqueous solution at 92–95 °C for 6 h, respectively, and then neutralized with Ba(OH)_2_ solution. After filtration, the solvent of the hydrolyzed solution was volatilized. The monosaccharides in the concentrated solution were identified by TLC with l-rhamnose and d-glucose references, which were developed with EtOAc-MeOH-HOAc-H_2_O (8:1:1:0.7) [3]. The *R_f_* values of l-rhamnose and d-glucose were 0.73 and 0.43, respectively.

### 3.5. Enzymatic Hydrolysis of Compound **5**

Compound **5** (20 mg) and cellulase (30 mg) were added to a 12 mL HOAc-NaOAc buffer solution (pH 5.0) and incubated at 37 °C for 6 h. The mixture solution was extracted with EtOAc and isolated on a silica gel column (EtOAc) to give (*E*,*R*)-3,7-dimethyl-oct-2-ene-1,6,7-triol and (*E*,*S*)-3,7-dimethyl-oct-2-ene-1,6,7-triol (6:4), which were affirmed by [α${]}_{D}^{27}$ +3.2 (*c* 0.19, EtOAc) [17].

### 3.6. Determination of Bioactivities

The bioactivities of compounds **1**–**8**, including FAS inhibitory action, α-glucosidase inhibitory action, α-amylase inhibitory action, DPPH radical scavenging capacity, and ABTS radical scavenging capacity, were measured in this study. The published methods [3,18,19,20] were employed in the biological tests, in which orlistat, acarbose, and l-(+)-ascorbic acid served as positive controls, respectively (Supplementary Materials S2).

### 3.7. Statistical Analyses

GraphPad Prism 5.01 was applied in statistical analyses. All compounds were measured in triplicate, and the results are reported as mean ± SD. The difference in means was determined by ANOVA with the statistical package SPSS 25.0. The significant difference between groups was confirmed at *p* < 0.05.

## 4. Conclusions

In conclusion, ten new monoterpenoid glycosides named ligurobustosides T_10_ (**1a**), T_11_ (**1b**), T_12_ (**2a**), T_13_ (**2b**), T_14_ (**3a**), T_15_ (**3b**), F_1_ (**4b**), T_16_ (**5a**), T_17_ (**5b**), and E_1_ (**6b**), together with five known ones (**4a**, **6a**, **7**, **8a**, **8b**), in which nine ingredients belonged to *trans*-*p*-hydroxycinnamic acid esters, were separated from the leaves of *L. robustum* and identified using the spectroscopic method (^1^H NMR, ^13^C NMR, ^1^H-^1^H COSY, HSQC, HMBC, NOEDS, HRESIMS, IR) and chemical method. The results of biological tests exhibited that the FAS inhibitory action of compound **5** (IC_50_: 4.38 ± 0.11 μM) was as strong as asorlistat (IC_50_: 4.46 ± 0.13 μM) used as a positive control; the α-glucosidase inhibitory actions of compounds **1**–**4** and **7**–**8,** and the α-amylase inhibitory actions of compounds **1**–**8** were medium and weaker than acarbose; the ABTS radical scavenging capacities of compounds **1**–**3** and **5**–**8** (IC_50_: 6.27 ± 0.23 ~ 8.59 ± 0.09 μM) were stronger than l-(+)-ascorbic acid (IC_50_: 10.06 ± 0.19 μM), a positive control. Together with our previous studies [3,15], monoterpenoid glycosides, phenylmethanoid glycosides, phenylethanoid glycosides, butenol and hexenol glycosides, sugar esters, and flavonoid glycosides, along with *trans*-*p*-hydroxycinnamic acid, were believed to be the main functional constituents of *L. robustum* to prevent diabetes and its complications. This research provided a theoretical foundation for the leaves of *L. robustum* to prevent diabetes and its complications. Nevertheless, the activities of the stereoisomer mixture might be different from those of the relevant pure compound, and the activities in vitro might be different from those in vivo. Thus, further research should be carried out to evaluate the activities of pure compounds in vivo in the future.

## Figures and Tables

**Table 1:** ^1^H NMR data of compounds **1**–**6** from the leaves of *L. robustum* in CD_3_OD *^a^*.

**No**	**1a *^b^***	**1b *^b^***	**2a *^c^***	**2b *^c^***	**3a *^c^***
1a	4.26 d (7.2)	4.26 d (7.2)	4.21 d (6.8)	4.21 d (6.8)	4.27 dd (12.0, 8.0)
1b	4.27 d (7.2)	4.27 d (7.2)	4.22 d (6.8)	4.22 d (6.8)	4.38 dd (8.0, 6.4)
2	5.37 t (7.2)	5.37 t (7.2)	5.35 t (6.8)	5.35 t (6.8)	5.40 dd (12.0, 6.4)
4	2.02 t (7.2)	2.03 t (7.2)	2.02 t (6.4)	2.02 t (6.4)	2.06 t (7.2)
5	1.48 m	1.48 m	1.48 m	1.48 m	1.51 m
6	1.40 m	1.40 m	1.40 m	1.40 m	1.41 m
8	1.15 s	1.15 s	1.16 s	1.16 s	1.19 s
9	1.15 s	1.15 s	1.16 s	1.16 s	1.19 s
10	1.66 s	1.66 s	1.64 s	1.64 s	1.70 s
Glc					
1′	4.32 d (7.8)	4.26 d (7.8)	4.28 d (8.0)	4.32 d (8.0)	4.38 d (7.6)
2′	3.30 m	3.30 m	3.29 m	3.30 m	3.40 m
3′	3.53 t (9.6)	3.48 t (9.6)	3.50 t (8.8)	3.51 t (8.8)	3.82 t (9.6)
4′	3.40 t (9.6)	3.38 t (9.6)	3.39 t (8.8)	3.38 t (8.8)	4.92 t (9.6)
5′	3.52 m	3.49 m	3.50 m	3.52 m	3.53 m
6′a	4.36 dd (12.0, 6.6)	4.29 dd (9.6, 6.6)	4.29 dd (12.0, 6.0)	4.35 dd (12.0, 6.0)	3.55 m
6′b	4.51 dd (12.0, 2.4)	4.48 dd (9.6, 1.8)	4.49 dd (12.0, 2.0)	4.50 dd (12.0, 2.0)	3.63 m
Rha					
1″	5.18 d (1.8)	5.15 d (1.8)	5.15 d (2.0)	5.17 d (2.0)	5.19 d (1.6)
2″	3.95 m	3.94 m	3.94 dd (3.2, 2.0)	3.94 dd (3.2, 2.0)	3.92 dd (3.2, 1.6)
3″	3.71 dd (9.6, 3.6)	3.70 dd (9.6, 3.6)	3.70 dd (9.6, 3.2)	3.70 dd (9.6, 3.2)	3.58 m
4″	3.40 t (9.6)	3.39 t (9.6)	3.39 t (9.6)	3.39 t (9.6)	3.29 m
5″	4.00 m	4.00 m	3.98 dd (9.6, 6.4)	4.00 dd (9.6, 6.4)	3.57 m
6″	1.25 d (6.6)	1.25 d (6.6)	1.24 d (6.4)	1.25 d (6.4)	1.09 d (6.4)
Cou					
2‴	7.45 d (8.4)	7.66 d (8.4)	7.65 d (8.8)	7.46 d (8.8)	7.47 d (8.4)
3‴	6.81 d (8.4)	6.74 d (8.4)	6.76 d (8.8)	6.81 d (8.8)	6.81 d (8.4)
5‴	6.81 d (8.4)	6.74 d (8.4)	6.76 d (8.8)	6.81 d (8.8)	6.81 d (8.4)
6‴	7.45 d (8.4)	7.66 d (8.4)	7.65 d (8.8)	7.46 d (8.8)	7.47 d (8.4)
7‴	7.64 d (16.2)	6.87 d (12.6)	6.88 d (12.8)	7.64 d (16.0)	7.66 d (16.0)
8‴	6.35 d (16.2)	5.76 d (12.6)	5.80 d (12.8)	6.31 d (16.0)	6.34 d (16.0)
**No**	**3b *^c^***	**4b *^c^***	**5a *^c^***	**5b *^c^***	**6b *^b^***
1a	4.27 dd (12.0, 8.0)	4.25 dd (6.4, 6.0)	4.26 d (7.2)	4.26 d (7.2)	4.26 d (7.2)
1b	4.38 dd (8.0, 6.4)	4.34 dd (12.0, 6.0)	4.27 d (7.2)	4.27 d (7.2)	
2	5.40 dd (12.0, 6.4)	5.38 dd (12.0, 6.4)	5.41 t (7.2)	5.41 t (7.2)	5.35 t (7.2)
4a	2.06 t (7.2)	2.07 m	2.08 m	2.08 m	2.03 t (7.2)
4b			2.29 m	2.29 m	
5a	1.51 m	1.63 m	1.33 m	1.38 m	2.09 m
5b			1.76 m	1.72 m	
6	1.41 m	3.97 t (6.0)	3.23 dd (10.4, 2.0)	3.22 dd (10.4, 2.0)	5.08 t (6.6)
8a	1.19 s	4.81 d (2.0)	1.12 s	1.11 s	1.66 s
8b		4.91 d (2.0)			
9	1.19 s	1.70 s	1.16 s	1.15 s	1.58 s
10	1.70 s	1.68 s	1.68 s	1.68 s	1.66 s
Glc					
1′	4.33 d (7.6)	4.30 d (8.0)	4.32 d (7.6)	4.32 d (7.6)	4.28 d (7.8)
2′	3.36 m	3.29 m	3.31 m	3.31 m	3.34 m
3′	3.75 t (9.6)	3.52 m	3.52 t (9.2)	3.52 t (9.2)	3.52 m
4′	4.86 t (9.6)	3.37 m	3.39 t (9.2)	3.39 t (9.2)	3.40 t (9.6)
5′	3.48 m	3.51 m	3.52 m	3.52 m	3.52 m
6′a	3.55 m	4.30 dd (12.0, 6.0)	4.36 dd (12.0, 6.0)	4.36 dd (12.0, 6.0)	4.30 dd (12.0, 6.0)
6′b	3.63 m	4.49 dd (12.0, 1.6)	4.51 dd (12.0, 2.0)	4.51 dd (12.0, 2.0)	4.45 br. d (12.0)
Rha					
1″	5.16 d (2.0)	5.15 d (2.0)	5.17 d (2.0)	5.17 d (2.0)	5.15 br. s
2″	3.92 dd (3.2, 2.0)	3.93 m	3.94 dd (3.2, 2.0)	3.94 dd (3.2, 2.0)	3.94 m
3″	3.58 m	3.70 dd (9.6, 3.2)	3.70 dd (9.6, 3.2)	3.70 dd (9.6, 3.2)	3.70 dd (9.6, 3.6)
4″	3.30 m	3.39 m	3.39 t (9.6)	3.39 t (9.6)	3.40 t (9.6)
5″	3.61 m	4.00 dd (9.6, 6.4)	4.00 dd (9.6, 6.4)	4.00 dd (9.6, 6.4)	4.00 dd (9.6, 6.0)
6″	1.16 d (6.4)	1.25 d (6.4)	1.24 d (6.4)	1.24 d (6.4)	1.25 d (6.0)
Cou					
2‴	7.72 d (8.4)	7.66 d (8.4)	7.46 d (8.4)	7.46 d (8.4)	7.64 d (8.4)
3‴	6.76 d (8.4)	6.74 d (8.4)	6.81 d (8.4)	6.81 d (8.4)	6.76 d (8.4)
5‴	6.76 d (8.4)	6.74 d (8.4)	6.81 d (8.4)	6.81 d (8.4)	6.76 d (8.4)
6‴	7.72 d (8.4)	7.66 d (8.4)	7.46 d (8.4)	7.46 d (8.4)	7.64 d (8.4)
7‴	6.95 d (12.8)	6.87 d (12.8)	7.64 d (16.0)	7.64 d (16.0)	6.87 d (13.2)
8‴	5.79 d (12.8)	5.77 d (12.8)	6.35 d (16.0)	6.35 d (16.0)	5.79 d (13.2)

*^a^* Coupling constants (*J* values in Hz) are reported in parentheses. *^b^* At 600 MHz. *^c^* At 400 MHz.

**Table 2:** ^13^C NMR data of compounds **1**–**6** from the leaves of *L. robustum* in CD_3_OD.

**No**	**1a *^a^***	**1b *^a^***	**2a *^a^***	**2b *^a^***	**3a *^b^***
1	66.2	66.2	66.2	66.2	66.5
2	121.3	121.4	121.3	121.3	121.5
3	142.4	142.4	142.5	142.5	142.2
4	41.0	41.0	41.0	41.0	41.0
5	23.3	23.4	23.4	23.4	23.4
6	44.2	44.2	44.3	44.3	44.2
7	71.3	71.4	71.4	71.4	71.4
8	29.2	29.2	29.2	29.2	29.2
9	29.2	29.2	29.2	29.2	29.2
10	16.4	16.4	16.4	16.4	16.4
Glc					
1′	102.4	102.4	102.4	102.4	102.6
2′	75.6	75.6	75.6	75.6	76.1
3′	84.0	84.5	84.1	84.0	81.6
4′	70.5	70.5	70.5	70.5	70.6
5′	75.6	75.5	75.3	75.6	76.1
6′	64.7	64.5	64.5	64.7	62.4
Rha					
1″	102.7	102.7	102.7	102.7	103.0
2″	72.3	72.3	72.3	72.3	72.3
3″	72.2	72.2	72.2	72.2	72.0
4″	73.9	74.0	74.0	74.0	73.7
5″	70.0	70.0	70.0	70.0	70.4
6″	17.9	17.9	17.9	17.9	18.4
Cou					
1‴	127.0	125.9	127.6	125.9	127.0
2‴	131.2	133.9	133.4	131.3	131.3
3‴	116.9	116.3	115.9	117.6	116.9
4‴	161.3	161.0	160.1	163.8	161.5
5‴	116.9	116.3	115.9	117.6	116.9
6‴	131.2	133.9	133.4	131.3	131.3
7‴	146.8	145.5	145.2	147.2	147.6
8‴	114.9	115.6	116.2	113.8	114.7
CO	169.0	168.2	168.1	169.3	168.3
**No**	**3b *^b^***	**4b *^a^***	**5a *^a^***	**5b *^a^***	**6b *^b^***
1	66.5	66.4	66.2	66.2	66.2
2	121.5	121.7	121.4	121.4	121.3
3	142.2	142.2	142.5	142.5	142.3
4	41.0	36.7	37.7	37.7	40.7
5	23.4	34.2	30.3	30.4	27.4
6	44.2	76.2	78.8	78.8	125.0
7	71.4	148.8	73.8	73.8	132.5
8	29.2	111.5	24.9	24.9	25.9
9	29.2	17.6	25.8	25.7	17.8
10	16.4	16.6	16.5	16.6	16.5
Glc					
1′	102.7	102.4	102.3	102.3	102.3
2′	76.0	75.6	75.6	75.6	75.6
3′	81.9	84.0	84.0	84.0	84.0
4′	70.7	70.5	70.5	70.5	70.5
5′	76.1	75.4	75.5	75.5	75.5
6′	62.4	64.5	64.7	64.7	64.9
Rha					
1″	103.1	102.8	102.7	102.7	102.7
2″	72.2	72.4	72.3	72.3	72.3
3″	72.1	72.3	72.2	72.2	72.2
4″	74.0	74.0	74.0	74.0	74.0
5″	70.0	70.0	70.0	70.0	70.0
6″	18.2	17.9	17.9	17.9	17.9
Cou					
1‴	127.5	127.0	127.1	127.1	127.6
2‴	134.3	133.9	131.2	131.2	133.8
3‴	115.8	116.3	116.9	116.9	115.9
4‴	160.4	161.4	161.5	161.5	160.1
5‴	115.8	116.3	116.9	116.9	115.9
6‴	134.3	133.9	131.2	131.2	133.8
7‴	147.3	145.5	147.8	147.8	145.3
8‴	115.8	115.6	114.9	114.9	116.2
CO	166.9	168.2	169.1	169.1	168.1

*^a^* At 150 MHz. *^b^* At 100 MHz.

**Table 3:** Results of the biological tests of compounds **1**–**8** from the leaves of *L. robustum ^a^*.

Compounds	FAS IC_50_ (μM) *^b^*	α-Glucosidase Inhibition at 0.1 mM (%)	α-Amylase Inhibition at 0.1 mM (%)	DPPH IC_50_ (μM) *^b^*	ABTS^•+^ IC_50_ (μM) *^b^*
**1**	NA *^c^*	56.6 ± 2.3 b	22.6 ± 2.4 b	NA	6.40 ± 0.34 a
**2**	>100	41.7 ± 3.3 c	29.8 ± 9.6 b	NA	7.52 ± 0.09 b
**3**	NA	34.6 ± 0.6 d	29.5 ± 2.8 b	NA	6.27 ± 0.23 a
**4**	24.68 ± 0.27 d	35.0 ± 2.4 d	28.4 ± 0.9 b	NA	12.54 ± 0.25 e
**5**	4.38 ± 0.11 a	NA	23.9 ± 3.6 b	NA	8.34 ± 0.19 c
**6**	9.78 ± 0.41 c	NA	33.3 ± 3.9 b	NA	8.59 ± 0.09 c
**7**	NA	20.8 ± 2.0 e	33.2 ± 2.7 b	NA	7.74 ± 0.10 b
**8**	6.78 ± 0.18 b	33.6 ± 3.4 d	30.2 ± 8.8 b	NA	7.34 ± 0.13 b
Orlistat *^d^*	4.46 ± 0.13 a				
Acarbose *^d^*		93.2 ± 0.1 a	51.8 ± 2.5 a		
l-(+)-ascorbic acid *^d^*				13.66 ± 0.13	10.06 ± 0.19 d

*^a^* Result is reported as the mean ± SD (*n* = 3). The means noted with different letters have significant differences (ANOVA, α = 0.05). *^b^* IC_50_: the eventual concentration of the tested compound needed to inhibit 50% of enzyme activity or clear away 50% of free radicals. *^c^* NA: no activity. *^d^* Positive control.

## Data Availability

The data presented in this study are available in the Supplementary Materials.

## Supplementary Materials

The following supporting information can be downloaded at: https://www.mdpi.com/article/10.3390/molecules28217274/s1, Figures S1–S6: ^1^H NMR, ^13^C NMR, ^1^H-^1^H COSY, HSQC, HMBC, NOEDS, HRESIMS, and IR spectra of compounds **1** (Figure S1), **2** (Figure S2), **3** (Figure S3), **4** (Figure S4), **5** (Figure S5), and **6** (Figure S6); S1: ^1^H NMR and ^13^C NMR data of compounds **4a**, **6a, 7**, **8a**, and **8b**; S2: determination of bioactivities.

## Sample Availability

Samples of the compounds are not available from the authors.

## Abbreviations

ABTS	2,2′-azino-bis(3-ethylbenzthiazoline-6-sulphonic acid) ammonium salt
Ac-CoA	acetyl-coenzyme A
ANOVA	one-way analysis of variance
CC	column chromatography
Cou	coumaroyl
DPPH	2,2-diphenyl-1-picrylhydrazyl
EtOAc	ethyl acetate
FAS	fatty acid synthase
Glc	glucosyl
^1^H-^1^H COSY	^1^H-^1^H homonuclear chemical shift correlation spectroscopy
HMBC	heteronuclear multiple bond coherence spectroscopy
HPLC	high-performance liquid chromatography
HRESIMS	high-resolution electrospray ionization mass spectroscopy
HSQC	heteronuclear single quantum coherence spectroscopy
IC_50_	half inhibitory concentration
IR	infrared absorption spectrum
Mal-CoA	methylmalonyl coenzyme A
NMR	nuclear magnetic resonance
NOEDS	nuclear Overhauser effect difference spectrum
Rha	rhamnosyl
SD	standard deviation
TLC	thin-layer chromatography
UV	ultraviolet–visible absorption spectrum
